# Insights into the Correlation between Toll-Like Receptor 2 Polymorphism and HBV-Related Disease Progression and Occurrence of Hepatocellular Carcinoma: A Case-Control Study in Egyptian Patients

**DOI:** 10.1155/2024/5797895

**Published:** 2024-07-19

**Authors:** Naglaa S. Elabd, Marwa L. Helal, Mohsen Elkhayat, Heba Kamal Abd-ElKhalek, Doaa M. Ahmed, Asmaa M. El-Shemy, Yara S. Elsaadawy, Rasha A. Abdelmoneum, Hind S. AboShabaan, Randa M. Seddik

**Affiliations:** ^1^ Department of Tropical Medicine Faculty of Medicine Menoufia University, Shebin El-Kom, Menoufia, Egypt; ^2^ Department of Clinical Biochemistry and Molecular Diagnostics National Liver Institute Menoufia University, Shebin El-Kom, Menoufia, Egypt; ^3^ Department of Internal Medicine Faculty of Medicine Menoufia University, Shebin El-Kom, Menoufia, Egypt; ^4^ Department of Radiology Faculty of Medicine Menoufia University, Shebin El-Kom, Menoufia, Egypt; ^5^ Department of Clinical Pathology Faculty of Medicine Menoufia University, Shebin El-Kom, Menoufia, Egypt; ^6^ Department of Medical Microbiology and Immunology Faculty of Medicine Ain Shams University, Cairo, Egypt; ^7^ Department of Clinical Oncology and Nuclear Medicine Faculty of Medicine Menoufia University, Shebin El-Kom, Menoufia, Egypt; ^8^ Biochemistry National Liver Institute Hospital Menoufia University, Shebin El-Kom, Menoufia, Egypt

## Abstract

**Methods:**

In total, 170 chronic HBV patients and 50 healthy controls of comparable age and gender were included in this case-control study. Clinical, laboratory, and imaging evaluations were conducted. ELISA was used to determine serum IL-6 levels, and TLR2 (rs3804099) genotyping allelic discrimination assay was performed using real-time PCR.

**Results:**

IL-6 values were significantly higher in the HCC group, followed by the cirrhotic group, than those in chronic hepatitis and control groups (*p* < 0.001), with a significant correlation with disease activity and progression parameters. TRL2 homozygous TT was the most frequent in the control group, but the CC genotype was significantly more prevalent in the HCC group than that in the other groups. Furthermore, the CC genetic variant was associated with higher levels of IL-6 and viral load in all HBV patients, whereas the TT genotype was associated with larger tumor size. Multivariate regression analysis demonstrated that in chronic HBV patients, viral load and TRL2 polymorphism are independent risk factors associated with the progression from chronic hepatitis to liver cirrhosis and to HCC. Similarly, the HBV viral load (*p*=0.03, OR = 2.45, and 95% CI: 1.69–3.65), IL-6 levels (*p*=0.04, OR = 3.45, and 95% CI: 2.01–6.9), and TRL2 variants (*p*=0.01, OR = 4.25, and 95% CI: 2.14–13.5) are independent risk factors associated with disease progression from cirrhosis to HCC.

**Conclusion:**

In chronic HBV patients, TRL2 polymorphism and higher IL-6 levels were positively correlated with a higher likelihood of HCC and chronic hepatitis B disease activity and progression.

## 1. Introduction

Hepatitis B infection is a common infectious disease of the liver caused by a hepatotropic virus known as the hepatitis B virus (HBV) [1]. Over two billion individuals have been infected with HBV globally, with over 290 million developing chronic HBV infections [2]. HBV infection is known to result in clinical manifestations that guarantee acute, fulminant, chronic, and occult forms. In the chronic form of HBV infection, the immune system fails to clear the virus from hepatocytes and sera [1]. Furthermore, prolonged HBV infections could induce liver cirrhosis and may alter cell conditions, causing hepatocellular carcinoma (HCC) [3].

Chronic hepatitis B (CHB) progression is substantially affected by host, viral, and environmental factors [4]. The interaction between viral and host genetic characteristics triggers viral infection susceptibility and disease progression, additionally influenced by metabolic, physiological, and environmental factors [5]. Han et al. discovered that HBV mutations significantly impact the effect of miRNA single nucleotide polymorphisms (SNPs) on vulnerability to HBV-associated hepatocellular carcinoma. Consequently, infections by a particular HBV genotype could be linked to host genetic polymorphisms. Furthermore, individual genetic predisposition may influence HCC development risk [6].

TLRs (toll-like receptors) are critical pattern recognition receptors. They have crucial functions in immune and inflammatory control, including antigen presentation, innate immune response and acquired immune response, and, most importantly, cytokine gene expression. TLR2 is indeed an outer membrane receptor that responds primarily to bacterial surface-associated pathogen-associated molecular patterns (PAMPs) in humans [7]. TLRs are thought to play a crucial role in the pathophysiology of a diversity of liver diseases because they are expressed in all types of liver cells, which comprise hepatic stellate cells (HSCs), Kupffer cells (KCs), and hepatocytes, as well as immune cells such as hepatic dendritic cells [8]. TLR-mediated inflammatory signaling pathways are linked to a variety of liver diseases, which include alcoholic and nonalcoholic liver disease, ischemia/reperfusion injury, hepatitis, liver fibrosis, cirrhosis, regeneration of the liver, and HCC [9].

Since TLRs are considered major molecules in immune responses to HBV infection, it was hypothesized that they also have essential functions in either eradicating HBV or inducing virus complications such as liver cirrhosis and HCC [10]. Thus, more research into the causal relationship between TLR genetic polymorphisms and the progression of HBV infection is required.

Variations in various cytokine activities have been observed during the course of HBV infections; however, an imbalance in proinflammatory and anti-inflammatory cytokine release impacts their immunopathogenesis [11]. Interleukin-6 (IL-6) is the primary mediator of inflammation and acute phase responses in the liver; it may also moderate chronic disease progression by safeguarding T cells from apoptosis during the inflammation process [12]. Additionally, IL-6 has pivotal roles in the malignant transformation of HCC progenitor cells and the growth of HCC [13].

Therefore, the purpose of the current research is to investigate the role of IL-6 and TLR2 gene polymorphism in chronic active hepatitis B and how they affect the progression of HBV-associated liver disease (liver cirrhosis and HCC).

## 2. Materials and Methods

### 2.1. Study Design and Participants

This case-control study was performed at Menoufia University's Departments of Tropical Medicine, Internal Medicine, Clinical Oncology, Radiology, and Clinical Pathology in collaboration with the Clinical Biochemistry and Molecular Diagnostics Department, National Liver Institute. In total, 220 participants were included in this study. They were chosen from Tropical Medicine, Internal Medicine, and Clinical Oncology departments' inpatient and outpatient clinics between January 2023 and May 2023. These participants were categorized into 58 HBV-linked chronic hepatitis patients, 62 patients with HBV-related cirrhosis, 50 patients with HCC on top of HBV infection, and 50 healthy controls of comparable age and gender.

Chronic hepatitis B was identified by the detection of HBsAg for more than six months, and liver cirrhosis was confirmed by clinical evaluations, laboratory investigations, and pelvi-abdominal ultrasonography. According to the WHO guidelines on the treatment of patients with CHB, the cutoff values for FibroScan were 7 to 8.5 kPa for the diagnosis of significant liver fibrosis and 11 to 14 kPa for the diagnosis of cirrhosis, respectively [14]. Moreover, triphasic CT abdomen was done to confirm HCC diagnosis (arterial enhancement with delayed washout). Patients with hepatic focal lesions other than HCC, those who had liver diseases other than HBV, such as chronic hepatitis C, autoimmune hepatitis, and metabolic hepatitis, and those with a history of hepatotoxic drug use or alcohol consumption were excluded from this study.

Patients and controls were subjected to a thorough history, clinical evaluation, and laboratory tests such as complete blood count, liver function tests, renal function tests, alpha-fetoprotein (AFP), and serological tests for viral markers by ELISA HBsAg, HIV, and HCV antibodies. Real-time PCR was conducted to assess the HBV viral load. Radiological evaluation was carried out for all participants using pelvi-abdominal ultrasonography, FibroScan for staging of fibrosis, and triphasic abdominal CT to confirm HCC diagnosis. Baseline computed tomography of the chest, abdomen, and pelvis and bone scans were performed to assess distant metastases in HCC patients. Moreover, the Child–Turcotte–Pugh (CTP) score was assessed, and esophagogastroduodenoscopy was done to determine the severity of liver disease and presence of gastroesophageal varices, respectively, in patients with liver cirrhosis and HCC.

## 3. Ethical Consideration

Following the development of the research questions and objectives, each participant was informed about the study and asked to provide informed consent before participating. The research was approved by the Research Ethics Committee, Faculty of Medicine, Menoufia University, Egypt (IRB: 1/2023TROP2), and was conducted in conformity with the Declaration of Helsinki.

## 4. Laboratory Investigations

In total, 10 mL of whole blood was collected from all participants from the cubital vein by venipuncture, 4 ml were collected into 2 EDTA tubes, one for immediate CBC measurement, then was centrifuged and plasma was preserved for IL6 assessment and the second was preserved at −80 till TLR2 SNP analysis. 2 mL was collected into sodium citrate tube for INR. The remaining 4 mL were collected into plain tube and centrifuged at high speed and the resultant serum was used to measure LFTs and AFP.

### 4.1. IL-6 Assay

IL-6 plasma level assessment: According to the manufacturer's instructions, the Human IL-6 ELISA Kit (enzyme-linked immunosorbent assay, Sigma-Aldrich, Germany, product number RAB0306) was used for measuring plasma IL-6 levels. Within thirty minutes, the results were read at 450 nm. The IL-6 concentrations in each sample were calculated after a standard curve was constructed.

## 5. Molecular Analysis

### 5.1. DNA Extraction

As per the manufacturer's instructions (Thermo Scientific, Lithuania, Gene jet whole blood genomic DNA purification Mini kit). The human genomic DNA has been extracted from whole venous blood collected in tubes containing EDTA by a spin column technique. Each qPCR reaction amplification volume is twenty *μ*L, composed of: 0.5 *μ*l of genotyping (primer/probe mix) assay, 10 *μ*L of genotyping qPCR Master Mix (2×) (Thermo Fisher Scientific, MA, USA), 3.5 *μ*L of DNAse-free water and 6 *μ*l (0.1 *μ*g/*μ*L) of genomic DNA. 6 *μ*L of DNAse-free water has been added to the negative control reaction.

### 5.2. Real-Time PCR

The PCR and genotyping have been carried out. The following cycling parameters were set: for 1 minute; holding stage at 60°C, at 95°C for 2 minutes' initial denaturation of genomic DNA occurred, 35 repeats of following: for 20 seconds denature at 94°C, for 20 seconds denature at 94°C, and for 20 seconds annealing at 55°C, for 30 seconds extension at 72°C. Furthermore, at 72°C for 2 minutes' final extension occurred and eventually holding stage for 1 minute at 60°C.

### 5.3. SNP Analysis of TLR2 Gene

The TLR-2 SNP identification: The TaqMan allelic discrimination assay was performed for rs3804099 genotyping. Using TaqMan 5′ nuclease assay chemistry for amplification and detection of specific SNP alleles variants in purified genomic DNA samples for the SNP Genotyping Assays. Every predesigned TaqMan SNP genotyping assay comprises 2 allele-specific TaqMan MGB probes that contains unique fluorescent dye as well as a PCR primer pair for identifying specific targets of SNP utilizing 96 well 7500 real-time fast PCR instrument (Thermo Fisher Scientific Inc., Life Technologies TM, CA, USA). To offer unmatched specificity for the allele of interest, these TaqMan probe and primer sets (assays) align in a unique way with the genome. Samples that contain both alleles 1 and 2 represented the heterozygotes. Meanwhile, samples containing the same allele (either allele 1 or allele 2) demonstrated the homozygotes.

### 5.4. Statistical Analysis

The data were collected and analyzed by SPSS (Statistical Package for Social Science) version 20.0 on an IBM-compatible computer (SPSS Inc., Chicago, IL, USA). Qualitative data were described as frequency and percentage and analyzed using the chi-square test, while quantitative data was represented as mean, standard deviation, median, and range and was analyzed between the four studied groups using the ANOVA test when the data were normally distributed. Kruskal–Wallis test was used to compare multiple groups with normally distributed data, and the post hoc test was employed to compare the groups in pairs. Spearman correlation was performed to correlate IL-6 and other quantitative parameters. The crude OR measured the risk of exposure to mutant genotypes. Regression analysis is a statistical process for estimating independent risk for progression of liver disease. The *P* value was considered significant when it was less than 0.05.

## 6. Results

The demographic, clinical, imaging, and upper endoscopy characteristics of the studied participants are summarized in Table 1. In total, 220 participants were included in this case-control study. There were 50 age- and gender-matched healthy controls (group I), 58 patients with chronic hepatitis B (group II) with a mean age of 54.57 ± 9.56 years, 62 patients with HBV-related liver cirrhosis (group III) with a mean age of 54.92 ± 10.88 years, and 50 patients with HBV-related HCC (group VI) with a mean age of 56.8 ± 8.87 years. A nonsignificant difference was observed between the studied groups in terms of age and gender (*p* value = 0.68 and 0.16, respectively). According to the clinical evaluation of studied patients, there were no significant differences observed between the cirrhotic and HCC groups in terms of the presence of jaundice, history of hepatic encephalopathy, ascites grade, and presence of esophageal varices in upper endoscopy (*p* value = 0.86, 0.76, 0.79, and 0.35, respectively).

Regarding radiological evaluation of the studied patients, fibrosis staging by FibroScan differed significantly between the three patient groups (*p* < 0.001), where, in chronic hepatitis B groups, F1, F2, and F3 were 15.5%, 55.2%, and 29.1%, respectively, and all cirrhotic patients (100%) were F4. However, in the HCC group, six (12%) patients were F3 and the rest (88%) were F4. The ultrasonography and triphasic CT findings in patients' groups are displayed in Table 1.

Concerning laboratory investigations, all parameters of complete blood count showed significant differences between the studied groups, with lower platelet counts were observed in the cirrhotic and HCC groups. Moreover, liver function tests and alpha-fetoprotein were significantly different among the four groups (*p* < 0.001 for all). HBV-DNA copies were higher in the cirrhotic group than those in other groups (*p*=0.002). IL-6 was found to have higher levels in the HCC group, followed by the cirrhotic group, than that in chronic hepatitis and control groups (*p* < 0.001), as shown in Table 2.

**Figure 1:**
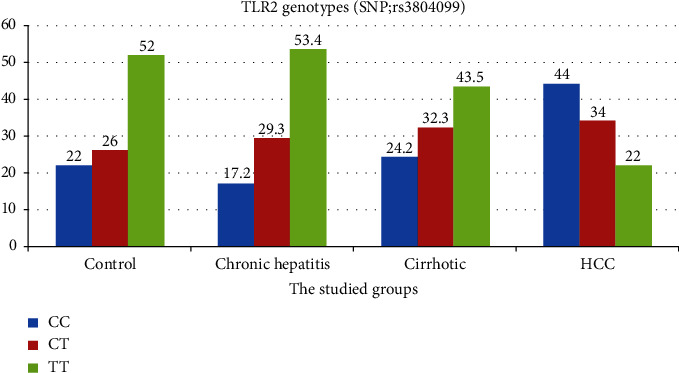
Genotypes frequencies for TLR-2 rs3804099 polymorphism among the studied groups.

Table 3 lists the genotype frequencies and allelic distribution of the TLR2 polymorphism (rs3804099) in the studied groups. When HBV patients were compared to control subjects, TRL2 rs3804099 genotype TT was the most frequent (52%) in the control group, followed by heterozygote mutant CT and CC mutant representing 26% and 22%, respectively; however, in HBV patients, the TT genotype was 53.4%, 43.5%, and 22% in chronic hepatitis, liver cirrhosis, and HCC groups, respectively. Moreover, the CC genotype was significantly more prevalent in the HCC group (44%) than that in the chronic hepatitis (17.2%) and cirrhotic (24.2%) groups (Figure 1). Additionally, the T allele was the most prevalent in control, chronic hepatitis, and cirrhotic groups, while the C allele represents the most prevalent one in the HCC group.

For risk assessment of TRL2 rs3804099 polymorphism in disease progression and HCC development, the statistical analysis revealed that homozygote CC and heterozygote CT variants and C allele are risk factors for disease progression from chronic HBV infection to HCC with odds ratio (OR) of 6.2 (95% CI: 2.25–17.12), OR = 2.82 (95% CI: 1.08–7.37), and OR = 3.34 (95% CI: 1.91–5.85), respectively. Furthermore, CC and CT genotypes and C allele are significant risk factors for disease progression from liver cirrhosis to HCC with OR of 3.60 (95% CI: 1.38–9.41), OR = 2.09 (95% CI: 0.80–5.41), and OR = 2.31 (95% CI: 1.35–3.97), respectively, as shown in Table 4.

Table 5 demonstrates the correlation between IL-6 values and the disease activity parameters in patients with chronic hepatitis B. We noticed that CHB patients experienced higher IL-6 levels significantly correlated with higher ALT, AST, viral load (HBV-DNA PCR), and a more advanced fibrosis stage (*r* = 0.35, *P*=0.008; *r* = 0.41, *P*=0.002; *r* = 0.35, *P*=0.007; *r* = 0.28, *P*=0.03, respectively). In cirrhotic patients, a significant positive correlation was observed between IL-6 levels and HBV-DNA PCR (*r* = 0.33, *P*=0.008). Moreover, in the HCC group, IL-6 displayed significant positive correlations with INR, serum creatinine, and AFP, together with significant negative correlations with serum albumin and platelet count (*r* = −0.34, *P*=0.02; *r* = −0.31, *P*=0.03).

**Figure 2:**
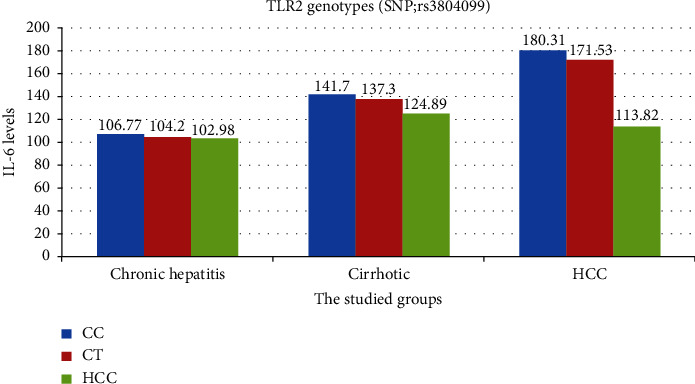
Correlation between rs3804099 genotypes of TLR2 and IL-6 profile among CHBV groups.

Table 6 demonstrates a significant association between TLR-2 genetic variants with parameters of disease activity and progression in chronic hepatitis and cirrhotic groups. For both groups, patients carrying the homogenous CC genetic variant had higher values of ALT, IL-6 (Figure 2), and viral load than the other variants. Additionally, as a marker of disease progression, the presence of the homogenous CC genetic variant is associated with higher mean values of total bilirubin and INR (*P*=0.02, 0.07), and with lower mean values of serum albumin (*P*=0.01).

According to Table 7, patients carrying the homogenous CC genetic variant of TLR-2 had higher values of IL-6 (Figure 2) and viral load (*P*=0.047 and 0.04, respectively). Additionally, those with the CT and CC variants had larger tumor sizes (*P*=0.03).

For the parameters influencing the susceptibility of disease progression in HBV-infected patients, multivariate regression analysis was applied. As demonstrated in model 2, the independent risk factors for progression from chronic hepatitis to HCC were HBV viral load and TRL2 rs3804099 polymorphism. Similarly, for progression from liver cirrhosis to HCC model 3, the HBV viral load (*p*=0.03, OR = 2.45, and 95% CI: 1.69–3.65), IL-6 levels (*p*=0.04, OR = 3.45, and 95% CI: 2.01–6.9), and TRL2 rs3804099 polymorphism (*p*=0.01, OR = 4.25, and 95% CI: 2.14–13.5) are the independent risk factors (Table 8).

## 7. Discussion

Liver cancer is the third leading cause of cancer-related death and ranks sixth as the most commonly diagnosed cancer. It accounts for 8.3% of cancer deaths overall and 4.7% of newly diagnosed cases. In 2021, liver cancer was responsible for approximately 30,000 deaths [15, 16]. Being the most common histological type of liver cancer, HCC accounts for more than 75% of all primary liver cancers worldwide [17], indicating its significant burden.

Because of the disease's poor prognosis, the incidence and mortality rates for HCC are roughly equal. The significant variation in HCC incidence and mortality rates throughout geographical areas could be explained by variations in environmental exposure, timing, and the prevalence of chronic viral hepatitis, access to healthcare resources, and the ability to detect and treat cancer early [18]. The majority of HCC cases are reported in individuals with underlying liver disease, the majority of which is caused by chronic hepatitis B and C virus infection or excessive consumption of alcohol. In addition to the viral and environmental risk factors, individual genetic predisposition may influence the risk of HCC development. Considerations of heritability and familial aggregations supported the influence of genetic factors [19–21].

Despite the immunization program's great success, a large number of patients have chronic HBV infection and, as a result, are at increased risk of cancer. Host immunity is indeed essential in the progression and HBV infection outcome [22]. In chronic HBV infection, IL-6 is a potent pleiotropic multifunctional inflammatory cytokine that is a crucial driver of hepatocyte repair and replication [23]. TLRs are a type of receptor that serves as the first line of defense against microbes. They are capable of recognizing both invading pathogens as well as endogenously dangerous molecules produced by damaged tissues and dying cells. Being expressed on DCs that link innate and adaptive immunity, TLRs serve as bridging molecules [24].

We investigated serum IL-6 and TRL2 rs3804099 gene distribution in healthy controls and HBV-infected patients. Furthermore, we explored the association between them and disease progression, liver function parameters, and risk of HCC development, as well as the tumor characteristics in HBV patients.

In the current study, IL-6 levels were considerably higher in the HCC group, followed by the cirrhotic group, than those in the chronic hepatitis and control groups. IL-6 exhibited a significant positive association with ALT, AST, and HBV-DNA copies in chronic hepatitis and HCC groups, as well as a significant positive correlation with serum creatinine in cirrhosis and HCC groups. Additionally, serum IL-6 was found to be significantly associated with AFP in HCC patients.

It has been reported that IL-6 is highly expressed in the early stages of both acute and chronic liver injury [25]. As an inflammatory mediator, IL-6 may contribute to the progression of HBV-associated liver cirrhosis [26]. Furthermore, Xia et al. previously demonstrated that the levels of IL-6 rise with the expression of hepatitis B Virus X protein (HBx) in the hepatocytes, and this could be explained by MyD88- (myeloid differentiation factor 88) dependent manner production of HBx-induced IL-6 [27].

In this research, we noticed that higher values of IL-6 were linked with higher levels of ALT and AST, pointing out the IL-6's role as an indicator of liver cell necrosis. Furthermore, IL-6 is positively associated with total bilirubin and INR while being negatively associated with serum albumin and platelets, implying that IL-6 might potentially be used as a marker for the progression of liver disease. These findings are consistent with those of previous studies that revealed a link between IL-6 levels and biochemical markers of liver disease [28, 29].

A meta-analysis study found that IL-6 concentrations were considerably higher in the HCC group than in healthy participants and patients with liver cirrhosis [30]. A four-marker model incorporating IGF2, IL-6, AFP, and platelet count could discriminate HCC from the liver with an AUC of 0.97 [31]. El-Folly et al. reported that high serum IL-6 levels could predict HCC development in chronic hepatitis B patients and that the diagnostic value of IL-6 improved when combined with AFP measurement. Additionally, they stated that the gathering of IL-6 and AFP values may provide new hope for earlier detection of HCC in HBV patients [32]. It was reported that IL-6 trans-signaling is required to promote the development of HCC by preventing DNA-damage-induced hepatocyte apoptosis and inducing endothelial cell proliferation, which in turn promotes tumor angiogenesis [33].

When HBV patients were compared to control subjects in terms of TRL2 rs3804099 gene distribution, we found that the CC homozygote and CT heterozygote gene variants were the least frequent in the controls. However, in HBV patients, both CC and CT gene variants are more prevalent, especially in HCC patients. Similarly, the T allele was the most common in controls, while the C allele was more prevalent in cirrhotic and HCC groups. Elbrolosy et al. found TRL2 polymorphism to be related to disease progression in chronic HBV infection in Egyptian patients, which is consistent with our findings. They reported that chronic HBV-infected patients with the mutant TT genotypes or heterozygous CT of TLR2 rs3804099 experienced less disease activity compared to those with the CC variant [29].

TLRs are a type of pattern recognition receptor family that serves as the foundation of the innate immune response [7]. Toll was discovered as a gene that controls the dorsal-ventral polarity of the *Drosophila* embryo; nevertheless, later studies revealed that it is implicated in antifungal immunity [34]. The discovery of these receptors increased the importance of the innate immune system. They can recognize both the external PAMPs and the internal damage-associated molecular patterns (DAMPs) [24].

TLRs can identify various viral components, such as nucleocapsids, envelope peptides, and nucleic acids, activate signaling pathways, and intensify the release of proinflammatory cytokines and interferons [35]. There is mounting evidence that cytokine-mediated immune responses have a substantial impact on the clinical outcomes of HBV infections [36]. Hepatitis B virus-mediated hepatic TLR2 signaling may postpone, but not preclude, HBV spread and persistent replication in hepatocytes. HBV infection can be detected and responded to by both the circulating and intrahepatic innate immune systems. Regrettably, the strong response is also responsible for hepatic necroinflammation, resulting severe liver damage [37].

This study identified substantial differences in genotype frequencies and allelic distribution for TLR rs3804099 among the studied groups. We discovered that the CC genotype and C allele were significantly more prevalent in the HCC group when compared to chronic hepatitis and cirrhotic groups, implying a role in HCC development. This is supported by multivariate regression analysis, revealing that TRL2 gene variants and HBV viral load are independent risk factors for the development of HCC in HBV-related chronic hepatitis and liver cirrhosis.

TLR2 has been linked to a variety of cancers. TLR2 (Delta22) polymorphisms were linked to an increased risk of gallbladder cancer [38], while TLR2 microsatellite GT polymorphism has been linked to sporadic colorectal cancer in Croatians [39]. Additionally, a link between TLR2 (−196 to −174 del) and cervical cancer susceptibility [40], as well as HCC development in HCV patients, was reported [41].

TLR2 rs3804099 is a synonymous SNP. For a long time, it was assumed that synonymous SNPs were insignificant because they did not change the polypeptide structure. Notwithstanding, since synonymous mutations were proven to be involved in diseases in numerous studies over the past decade, this concept has changed. The following mechanisms may be involved in the effect on gene function: mRNA structure, mRNA stability, mRNA splicing changes, and protein folding [42].

Although TLRs are important components of the innate immune system and play an important role in the host-defensive mechanism against microbes, overactivation of TLRs could disrupt immune homeostasis, resulting in excessive proinflammatory cytokine production, which is undoubtedly a contributing factor to the pathogenesis of several inflammatory and autoimmune diseases [42]. Thereby, TLR signaling pathway inhibition is expected to be a beneficial therapeutic strategy for suppressing undesired, disease-linked inflammatory responses, which can be achieved using two main methods: blocking TLR ligand binding to the receptor or interrupting with intracellular signaling pathways to halt signal transduction [43]. Furthermore, some clinical trials indicated that therapeutic manipulation of TLR pathways could serve as a novel strategy for reversing chronic liver diseases [44].

This study has some limitations, as follows. First, this is a cross-sectional study, which does not allow us to follow up on the same patients at different stages of HBV infection. Therefore, a longitudinal study could aid in more precise detection of the actual impact of IL-6 levels and TLR2 rs3804099 SNP variation on liver disease progression and outcome in HBV patients. Second, the study has small sample size; thus, larger studies are warranted to validate our results.

## 8. Conclusion

The current study revealed that TLR2 rs3804099 polymorphism and IL-6 were positively correlated with CHB disease activity and progression. Therefore, they may be performed practically to assess the disease progression in CHB patients and those who are more susceptible to developing HCC.

## Figures and Tables

**Table 1:** Comparison between the studied groups regarding clinical data, ultrasound, CT scanning, and upper endoscopy.

Variables	Control *N* = 50	Chronic hepatitis *N* = 58	Cirrhosis *N* = 62	HCC *N* = 50	*P* value
Age (years)					
Mean ± SD	55.3 ± 10.67	54.57 ± 9.56	54.92 ± 10.88	56.8 ± 8.87	*F* = 0.50
Median (min.–max.)	55 (37–73)	54 (32–71)	56 (37–73)	57 (44–70)	*P* value = 0.68
Gender					
Male	42 (84.0)	40 (75.9)	45 (72.6)	44 (88.0)	*X* ^2^ = 5.14
Female	8 (16.0)	14 (24.1)	17 (27.4)	6 (12.0)	*P* value = 0.16
Jaundice					
Yes	—	—	14 (22.6)	12 (24)	*X* ^2^ = 0.03
No	—	—	48 (77.4)	38 (76)	*P* value = 0.86
Encephalopathy					
Yes	—	—	19 (30.6)	14 (28)	*X* ^2^ = 0.09
No	—	—	43 (69.4)	36 (72)	*P* value = 0.76
Ascites					
No	—	—	43 (69.4)	37 (74.0)	*X* ^2^ = 0.451
Mild/moderate	—	—	15 (24.2)	11 (22.0)	*P* value = 0.79
Marked ascites	—	—	4 (6.4)	2 (4.0)	
Child-Paugh score					
A	—	—	26 (41.9)	35 (70.0)	*X* ^2^ = 9.10
B	—	—	27 (43.5)	10 (20.0)	*P* value = 0.01
C	—	—	9 (14.5)	5 (10.0)	
Gastroesophageal varices					
No	—	—	40 (64.5)	28 (56.0)	*X* ^2^ = 0.8416
Yes	—	—	22 (35.5)	22 (44.0)	*P* value = 0.35
FibroScan finding					
F1	—	9 (15.5)	0 (0.0)	0 (0.0)	*X* ^2^ = 153.9
F2	—	32 (55.2)	0 (0.0)	0 (0.0)	*P* value <0.001
F3	—	17 (29.1)	0 (0.0)	6 (12.0)	
F4	—	0 (0.0)	62 (100)	44 (88.0)	
Liver by U/S and/or CT					
Liver size					
Normal size		28 (48.3)	16 (25.8)	12 (24)	*X* ^2^ = 45.26
Hepatomegaly		30 (51.7)	12 (19.4)	17 (34)	*P* value <0.001
Shrunken liver		0 (0.0)	34 (54.8)	21 (42)	
Echogenicity					
Normal		58 (100)	0 (0.0)	3 (6)	*X* ^2^ = 157.7
Cirrhosis		0 (0.0)	62 (100)	47 (94)	*P* value <.001
Portal vein diameter					
Normal		58 (100)	26 (41.9)	21 (42)	*X* ^2^ = 54.5
Dilated		0 (0.0)	36 (58.1)	29 (58)	*P* value <0.001
Portal vein patency					
Patent		58 (100)	61 (98.6)	43 (86)	*X* ^2^ = 14.07
Thrombosed		0 (0)	1 (1.6)	5 (10)	*P* value = 0.007
Malignant invasion		0 (0.0)	0 (0.0)	2 (4)	
Hepatic focal lesion (s)		—	—		
Size					
(<5 cm)				31 (62)	
(>5 cm)				19 (38)	
Number					
Single				29 (58)	
Multiple				21 (42)	
Spleen size by U/S					
Normal		44 (75.9)	22 (35.5)	10 (20.0)	*X* ^2^ = 37.26
Splenomegaly		14 (24.1)	40 (64.5)	40 (80.0)	*P* value <0.001

**Table 2:** Comparison between the studied groups regarding laboratory parameters and serum IL-6 level.

Variables	Control *N* = 50	Chronic hepatitis *N* = 58	Cirrhosis *N* = 62	HCC *N* = 50	*P* value
Platelets (×1000/*μ*l)					
Mean ± SD	281.18 ± 67.12	232.5 ± 39.33	175.74 ± 48.14	135.5 ± 63.27	*F* = 70.0
Median (min.–max.)	256 (174–400)	232 (187–301)	181 (89–260)	108.5 (71–264)	*P* value <0.001
Post hoc		<0.001^1^	<0.001^1^ <0.001^2^	<0.001^1^ <0.001^2^ <0.001^3^	
Hb (gm/dl)					
Mean ± SD	13.86 ± 0.85	13.54 ± 1.18	12.96 ± 1.54	13.62 ± 1.58	*F* = 4.72
Median (min.–max.)	14 (12.5–15)	13.2 (11.8–16)	12.6 (11.3–16.6)	13.55(10.9–16.4)	*P* value = 0.003
Post hoc		0.21^1^	<0.001^1^ 0.02^2^	0.37^1^ 0.75^2^ 0.01^3^	
WBCs (×1000/*μ*l)					
Mean ± SD	7.57 ± 2.0	6.55 ± 1.22	5.99 ± 1.27	6.06 ± 1.23	*F* = 13.33
Median (min.–max.)	7.75 (4.5–11)	6.4 (4.9–9.3)	6.1 (3.7–8.4)	6 (4.4–8.3)	*P* value <0.001
Post hoc		<0.001^1^	<0.001^1^ 0.03^2^	<0.001^1^ 0.08^2^ 0.80^3^	
ALT (IU/L)					
Mean ± SD	17.72 ± 3.87	119.67 ± 51.39	97.47 ± 57.46	60.0 ± 19.04	*K* = 151
Median (min.–max.)	18 (12–24)	99 (71–241)	75 (49–220)	52.5 (37–96)	*P* value <0.001
Post hoc		<0.001^1^	<0.001^1^ 0.15^2^	<0.001^1^ <0.001^2^ <0.001^3^	
AST (IU/L)					*K* = 122
Mean ± SD	19.76 ± 4.14	93.62 ± 39.88	109.84 ± 69.71	72.94 ± 22.24	*P* value <0.001
Median (min.–max.)	19 (13–26)	85 (56–210)	81.5 (57–274)	68 (42–113)	
Post hoc		<0.001^1^	<0.001^1^ 0.53^2^	<0.001^1^ <0.006^2^ 0.001^3^	
Total bilirubin (mg/dl)					
Mean ± SD	0.82 ± 0.11	0.91 ± 0.30	1.40 ± 0.84	1.32 ± 0.54	*K* = 39.83
Median (min.–max.)	0.8 (0.7–1)	0.9 (0.4–1.6)	1.1 (0.4–3.4)	1.25(0.6–2.4)	*P* value <0.001
Post hoc		0.23^1^	<0.001^1^ <0.001^2^	<0.001^1^ <0.001^2^ 0.99^3^	
Direct bilirubin (mg/dl)					*K* = 79.49
Mean ± SD	0.25 ± 0.050.3 (0.2–0.3)	0.51 ± 0.21	0.76 ± 0.49	0.78 ± 0.39	*P* value <0.001
Median (min.–max.)		0.5 (0.09–0.9)	0.8 (0.1–1.9)	0.74 (0.2–1.2)	
Post hoc		<0.001^1^	<0.001^1^ 0.001^2^	<0.001^1^ 0.002^2^ 0.99^3^	
Albumin (gm/dl)					*F* = 100.83
Mean ± SD	4.86 ± 0.25	4.16 ± 0.22	3.37 ± 0.71	4.47 ± 0.60	*P* value <0.001
Median (min.–max.)	4.9 (4.5–5.3)	4.1 (3.8–4.5)	3.4 (2.3–4.5)	3.45 (2.4–4.5)	
Post hoc		<0.001^1^	<0.001^1^ <0.001^2^	<0.001^1^ <0.001^2^ 0.31^3^	
INR					*F* = 81.08
Mean ± SD	0.91 ± 0.09	1.04 ± 0.06	1.29 ± 0.22	1.36 ± 0.24	*P* value <0.001
Median (min.–max.)	0.9 (0.8–1)	1 (0.9–1.1)	1.2 (1–1.7)	1.3 (1–1.8)	
Post hoc		<0.001^1^	<0.001^1^ <0.001^2^	<0.001^1^ <0.001^2^ 0.03^3^	
Creatinine (mg/dl)					
Mean ± SD	0.89 ± 0.14	0.66 ±0 .0.14	0.82 ± 0.18	0.76 ± 0.21	*F* = 18.13
Median (min.–max.)	0.9 (0.7–1.1)	0.68 (0.4–0.9)	0.8 (0.56–1.2)	0.68 (0.5–1.1)	*P* value <0.001
Post hoc		<0.001^1^	0.01^1^ <0.001^2^	<0.001^1^ 0.003^2^ 0.09^3^	
HBV- DNA copies (Log10)					*F* = 12.76
Mean ± SD		4.05 ± 0.27	4.27 ± 0.56	3.78 ± 0.62	*P* value <0.001
Median (min.–max.)		4.04 (3.53–4.55)	4.13 (3.58–5.52)	3.87 (2.54–4.62)	
Post hoc			0.02^1^	0.006^1^ <0.001^2^	
AFP (IU/ml)					*F* = 170.13
Mean ± SD	2.68 ± 1.11	5.03 ± 2.94	11.29 ± 5.44	3907.0 ± 4383.4	*P* value <0.001
Median (min.–max.)	3 (1–4)	4.09 (1.8–11.8)	10 (2.3–25.6)	2705(180–15110)	
Post hoc		<0.001^1^	<0.001^1^ <0.001^2^	<0.001^1^ <0.001^2^ <0.001^3^	
IL-6 (pg/mL)					*F* = 143.71
Mean ± SD	15.0 ± 3.29	105.33 ± 4.94	132.97 ± 13.93	162.7 ± 71.02	*P* value <0.001
Median (min.–max.)	14.5 (10–21)	105 (95–115)	136.5 (110–160)	180 (83–288)	
Post hoc		<0.001^1^	<0.001^1^ <0.001^2^	<0.001^1^ <0.001^2^ 0.03^3^	

Hb: hemoglobin concentration, WBCs: white blood cells, ALT: alanine transaminase, AST: aspartate transaminase, PT: prothrombin time, INR: international normalized ratio, IL-6: interleukin-6, SD: Standard deviation, *F*: ANOVA test, *K*: Kruskal–Wallis test, and *χ*^2^: chi-square test. 1 = comparison with the control group; 2 = comparing with chronic hepatitis; 3 = comparison with cirrhotic group.

**Table 3:** Genotypes frequencies and allelic distribution for TLR-2 polymorphism among the studied groups.

	Control *N* = 50	Chronic hepatitis *N* = 58	Cirrhotic *N* = 62	HCC *N* = 50	*χ* ^2^	*P* value
TLR-2 genotypes						

CC	11 (22.0)	10 (17.2)	15 (24.2)	22 (44.0)	16.34	0.01
CT	13 (26.0)	17 (29.3)	20 (32.3)	17 (34.0)		
TT	26 (52.0)	31 (53.4)	27 (43.5)	11 (22.0)		

TLR-2 alleles	*N* = 100	*N* = 116	*N* = 124	*N* = 100		

C	35 (35.0)	37 (31.9)	50 (40.3)	61 (61.0)	21.87	<0.001
T	65 (65.0)	79 (68.1)	74 (59.7)	39 (39.0)		

TLR-2: toll-like receptor-2; *χ*^2^: chi-square test.

**Table 4:** Risk assessment (odds ratio) of TLR-II polymorphism between the HCC group and both chronic hepatitis and cirrhotic groups.

	Chronic hepatitis# *N* = 58	HCC 5	*χ* ^2^ (p)	Odds ratio (95% CI)
TLR2 genotypes				

CC	10 (17.2)	22 (44.0)	13.31(<0.001)	6.2 (2.25–17.12)
CT	17 (29.3)	17 (34.0)	4.58 (0.03)	2.82 (1.08–7.37)
TT**∗**	31 (53.4)	11 (22.0)	—	—

TLR2 alleles	*N* = 116	*N* = 100		

C	37 (31.9)	61 (61.0)	18.35 (<0.001)	3.34 (1.91–5.85)
T**∗**	79 (68.1)	39 (39.0)	—	—

	Cirrhotic# *N* = 62	HCC *N* = 50	Test (p)	Odds ratio (95% CI)

TLR2 genotypes				
CC	15 (24.2)	22 (44.0)	7.08 (0.008)	3.60 (1.38–9.41)
CT	20 (32.3)	17 (34.0)	2.32 (0.13)	2.09 (0.80–5.41)
TT^*∗*^	27 (43.5)	11 (22.0)	—	—
TLR2 alleles	*N* = 124	*N* = 100		

C	50 (40.3)	61 (61.0)	9.47 (0.002)	2.31 (1.35–3.97)
T^*∗*^	74 (59.7)	39 (39.0)	—	—

TLR-2: toll-like receptor-2; ^*∗*^reference category; #: reference group; CI: confidence interval.

**Table 5:** Correlation between IL-6 profile and disease activity parameters among CHBV groups.

Variables	Chronic hepatitis (*n* = 58)		Cirrhotic (*n* = 62)		HCC (*n* = 50)	
	*r*	*P* value	*r*	*P* value	*r*	*P* value
Age	0.20	0.13	0.37	0.003	0.14	0.33
ALT (IU/L)	0.35	0.008	0.14	0.29	0.31	0.03
AST (IU/L)	0.41	0.002	0.06	0.66	0.30	0.03
Total bilirubin (mg/dl)	0.37	0.0.005	0.20	0.12	−0.09	0.52
Direct bilirubin (mg/dl)	0.17	0.20	0.07	0.59	−0.10	0.48
Albumin (gm/dl)	−0.35	0.007	−0.24	0.06	−0.34	0.02
HBV-DNA copies (IU/ml)	0.35	0.007	0.33	0.008	0.32	0.02
INR	−0.05	0.72	0.15	0.26	0.62	<0.001
Platelet count (X 10^3^)	−0.06	0.66	−0.18	0.15	−0.31	0.03
Hb (gm/dl)	0.41	0.002	0.39	0.002	−0.28	0.05
WBCs (X 10^3^)	−0.07	0.61	−0.13	0.30	0.12	0.42
Creatinine (mg/dl)	0.28	0.04	0.38	0.001	0.45	0.001
AFP (IU/ml)	−0.02	0.86	0.19	0.14	0.81	<0.001
Fibrosis stage	−0.22	0.09	0.0	1.0	0.0	1.0
Liver by U/S	0.41	0.001	0.0	1.0	0.0	1.0
Child-Pugh score	—	—	0.23	0.07	0.18	0.20

ALT: alanine transaminase; AST: aspartate transaminase; PT: prothrombin time; INR: international normalized ratio; Hb: hemoglobin concentration; WBCs: white blood cells; IL-6: interleukin-6.

**Table 6:** Correlation between rs3804099 genotypes of TLR2 and disease activity parameters and IL-6 profile among Chronic Hepatitis B and cirrhosis groups.

TLR2 rs3804099 genotypes Chronic hepatitis (*N* = 58)					
	CC *N* = 10	CT *N* = 17	TT *N* = 31	*K*	*P* value

ALT					
Mean ± SD	131.26 ± 61.50	109.53 ± 34.14	101.0 ± 29.17	1.13	0.57
Median (range)	122 (71–241)	99 (78–212)	90 (71–158)		
AST					
Mean ± SD	69.0 ± 17.8	98.41 ± 44.76	98.9 ± 40.2	8.66	0.01
Median (range)	60.0 (56–102)	79 (60–210)	90 (56–210)		
Viral load					
Mean ± SD	3.83 ± 0.31	4.16 ± 0.26	4.07 ± 0.23	7.42	0.02
Median	3.75	4.15	4.04		
Range	3.53–4.48	3.75–4.55	3.53–4.55		
IL-6					
Mean ± SD	106.77 ± 5.0	104.2 ± 4.26	102.98 ± 4.77	4.88	0.10
Median (range)	105 (100–115)	102.0 (100–110)	101.5 (95–110)		
Fibrosis					
Stage 1	0 (0.0)	3 (17.6)	6 (19.4)	*X* ^2^	
Stage 2	7 (80.0)	9 (52.9)	16 (48.4)	2.38	0.67
Stage 3	3(20.0)	5 (29.4)	9 (32.3)		
Stage 4	0 (0.0)	0 (0.0)	0 (0.0)		

TLR2 rs3804099 genotypes cirrhosis (*N* = 62)					
	CC *N* = 15	CT *N* = 20	TT *N* = 27	*K*	*P* value

ALT					
Mean ± SD	105.46 ± 60.41	88.60 ± 46.36	99.59 ± 64.10	0.01	0.99
Median (range)	75 (49–186)	75 (49–215)	68 (49–220)		
AST					
Mean ± SD	114.67 ± 66.58	92.2 ± 43.44	120.22 ± 85.36	0.41	0.81
Median (range)	84 (57–210)	84 (57–203)	79 (57–274)		
Viral load					
Mean ± SD	4.64 ± 0.81	4.29 ± 0.40	4.05 ± 0.38	7.64	0.02
Median	4.38	4.16	4.03		
Range	3.5 –5.52	3.58–5.0	3.58–4.89		
IL-6					
Mean ± SD	141.7 ± 9.18	137.3 ± 14.28	124.89 ± 10.70	15.57	<0.001
Median (range)	142 (119–160)	145 (110–150)	120 (110–145)		
Serum albumin					
Mean ± SD	3.09 ± 0.88	3.21 ± 0.63	3.66 ± 0.57	8.72	0.01
Median (range)	2.6 (2.3–4.5)	3.1 (2.6–4.5)	3.8 (2.7–4.5)		
Total bilirubin					
Mean ± SD	2.09 ± 1.09	1.11 ± 0.62	1.24 ± 0.61	7.61	0.02
Median (range)	2 (0.7–3.4)	0.99 (0.4–2.1)	1.1 (0.4–2.7)		
INR					
Mean ± SD	1.40 ± 0.29	1.29 ± 0.18	1.23 ± 0.28	5.36	0.07
Median (range)	1.5 (1–1.7)	1.3 (1–1.6)	1.2 (1–1.7)		

*K*: Kruskal–Wallis test.

**Table 7:** Correlation between rs3804099 genotypes of TLR2 and disease activity parameters and IL-6 profile in HCC group.

TLR2 rs3804099 genotypes HCC (*N* = 50)					

	CC *N* = 22	CT *N* = 17	TT *N* = 11	*K*	*P* value

ALT					
Mean ± SD	54.36 ± 17.87	66.71 ± 23.26	60.91 ± 9.67	4.68	0.10
Median (range)	46 (37–96)	52 (37–96)	65 (48–71)		
AST					
Mean ± SD	68.68 ± 21.76	77.82 ± 27.82	73.91 ± 10.47	2.80	0.25
Median (range)	55 (42–113)	60 (42–113)	70 (59–90)		
Viral load					
Mean ± SD	3.55 ± 0.70	3.84 ± 0.55	4.16 ± 0.33	7.44	0.02
Median	3.7	3.92	4.04		
Range	2.54–4.62	3.04–4.62	3.82–4.62		
IL-6					
Mean ± SD	180.31 ± 73.18	171.53 ± 70.43	113.82 ± 45.73	6.12	0.047
Median (range)	185 (83–288)	180 (90–288)	100 (83–220)		
Serum albumin					
Mean ± SD	3.19 ± 0.62	3.61 ± 0.31	3.81 ± 0.69	8.28	0.02
Median (range)	3.2 (2.4–4.5)	3.5 (3–4.1)	4 (2.9–4.5)		
Total bilirubin				1.08	0.058
Mean ± SD	1.38 ± 0.46	1.34 ± 0.74	1.17 ± 0.22		
Median (range)	1.25 (0.6–2.1)	1.1 (0.6–2.4)	13 (0.9–1.5)		
INR				2.51	0.29
Mean ± SD	1.43 ± 0.27	1.34 ± 0.14	1.26 ± 0.25		
Median (range)	1.5 (1–1.8)	1.3 (1.2–1.6)	1.3 (1–1.8)		
Child-Paugh					
A	13 (59.1)	12 (70.6)	10 (90.9)	7.28	0.12
B	5 (22.7)	5 (29.4)	0 (0.0)		
C	4 (18.2)	0 (0.0)	1 (9.1)		
Lesion size					
Mean **±** SD	4.74 **±** 2.11	6.90 ± 3.50	4.10 ± 3.73	6.97	0.03
Range	1.3–9.88	1.3–11.2	1.3–9.88		

**Table 8:** Multivariate regression analysis for independent risk factors affecting the progression of liver disease.

	SE	Wald *X*^2^	*P* value	Odds ratio	95% CI
*Model 1*					
Viral load	0.67	0.02	0.91	1.12	0.06–2.55
Fibrosis	2.69	0.31	0.65	0.99	0.18–4.15
IL-6	1.11	0.15	0.74	1.06	0.51–5.12
TLR2	1.81	0.41	0.42	0.98	0.21–2.58

*Model 2*					
Viral load	2.33	2.11	0.03	2.58	1.66–10.5
Fibrosis	1.58	1.33	0.18	1.11	0.66–3.65
IL-6	0.69	0.12	0.88	0.95	0.08–8.14
TLR2	1.16	2.56	0.01	3.12	2.01–11.45

*Model 3*					
Viral load	1.22	3.87	0.03	2.45	1.69–3.65
Fibrosis	3.13	1.32	0.19	1.08	0.66–1.66
IL-6	2.58	3.36	0.04	3.45	2.01–6.9
TLR2	1.36	5.90	0.01	4.25	2.14–13.5

SE: standard error; CI: confidence interval. Model 1: independent risk factors for progression from chronic hepatitis to cirrhotic group. Model 2: independent risk factors for progression from chronic hepatitis to HCC group. Model 3: independent risk factors for progression from the cirrhotic group to the HCC group.

## Data Availability

All data generated or analyzed during this study are included in this published article.

## Abbreviations

AFP: *α*-Fetoprotein
ALT: Aspartate transaminase
AST: Alanine transaminase
DNA: Deoxyribonucleic acid
HbsAg: Hepatitis B surface antigen
HB: Hemoglobin concentration
HBV: Hepatitis B virus
HCV: Hepatitis C virus
HIV: Human immunodeficiency virus
ELISA: Enzyme-linked immunosorbent assay
INR: International normalized ratio
WBCs: White blood cells.
